# Inulin Reduces Kidney Damage in Type 2 Diabetic Mice by Decreasing Inflammation and Serum Metabolomics

**DOI:** 10.1155/2024/1222395

**Published:** 2024-05-02

**Authors:** Jiayuan He, Xiang Li, Man Yan, Xinsheng Chen, Chang Sun, Jiajun Tan, Yinsheng Song, Hong Xu, Liang Wu, Zhengnan Yang

**Affiliations:** ^1^Health Testing Center, Zhenjiang Center for Disease Control and Prevention, Zhenjiang 212002, China; ^2^Medical Laboratory Department, Huai'an Second People's Hospital, Huai'an 223022, China; ^3^Department of Laboratory Medicine, School of Medicine, Jiangsu University, Zhenjiang 212013, China; ^4^Hospital Infection-Disease Control Department, Zhenjiang First People's Hospital, Zhenjiang 212002, China; ^5^Department of Clinical Laboratory, Yizheng Hospital, Nanjing Drum Tower Hospital Group, Yizheng 210008, China

**Keywords:** inflammation, inulin, metabolomics, mechanism, renal injury, type 2 diabetes (T2DM)

## Abstract

This study is aimed at assessing the impact of soluble dietary fiber inulin on the treatment of diabetes-related chronic inflammation and kidney injury in mice with type 2 diabetes (T2DM). The T2DM model was created by feeding the Institute of Cancer Research (ICR) mice a high-fat diet and intraperitoneally injecting them with streptozotocin (50 mg/kg for 5 consecutive days). The thirty-six ICR mice were divided into three dietary groups: the normal control (NC) group, the T2DM (DM) group, and the DM + inulin diet (INU) group. The INU group mice were given inulin at the dose of 500 mg/kg gavage daily until the end of the 12th week. After 12 weeks, the administration of inulin resulted in decreased serum levels of fasting blood glucose (FBG), low-density lipoprotein cholesterol (LDL-C), blood urea nitrogen (BUN), and creatinine (CRE). The administration of inulin not only ameliorated renal injury but also resulted in a reduction in the mRNA expressions of inflammatory factors in the spleen and serum oxidative stress levels, when compared to the DM group. Additionally, inulin treatment in mice with a T2DM model led to a significant increase in the concentrations of three primary short-chain fatty acids (SCFAs) (acetic acid, propionic acid, and butyric acid), while the concentration of advanced glycation end products (AGEs), a prominent inflammatory factor in diabetes, exhibited a significant decrease. The results of untargeted metabolomics indicate that inulin has the potential to alleviate inflammatory response and kidney damage in diabetic mice. This beneficial effect is attributed to its impact on various metabolic pathways, including glycerophospholipid metabolism, taurine and hypotaurine metabolism, arginine biosynthesis, and tryptophan metabolism. Consequently, oral inulin emerges as a promising treatment option for diabetes and kidney injury.

## 1. Background

Type 2 diabetes (T2DM) is a chronic metabolic disease affected by multiple factors. It is also one of the most common diseases globally, mainly manifested by persistent hyperglycemia [1]. According to statistics from the World Health Organization (WHO), the prevalence of T2DM has sharply increased in countries with various income levels over the past 3 years. Currently, there are approximately 422 million people worldwide with diabetes, with the majority living in low-income and middle-income countries. Every year, 1.5 million deaths are directly attributed to diabetes. With the improvement in living quality and lifestyle, the prevalence rate of adult diabetes in China has increased rapidly, and the number of patients has reached 120 million [2]. Complications caused by diabetes have brought severe economic burdens and health hazards to patients [3]. Diabetic nephropathy is a serious microvascular complication of diabetes and also the leading cause of chronic renal failure and end-stage renal disease [4]. However, the pathogenesis of diabetic nephropathy has not been fully elucidated, and there is still a lack of specific treatment for diabetic nephropathy. Diabetic nephropathy mainly involves systemic chronic inflammatory reaction, high oxidative stress response, and polyol pathway activation [5]. Long-term chronic inflammatory response and high oxidative stress response caused by high blood sugar and advanced glycation end products (AGEs) play a crucial role in the occurrence and development of diabetic nephropathy [6]. AGEs are a class of isomeric molecules formed by nonenzymatic glycosylation of biological macromolecules (proteins, lipids, or nucleic acids) with reducing sugars such as glucose [7]. Once the AGE generation reaction occurs, this reaction is almost irreversible, and the hyperglycemic environment in T2DM patients accelerates the generation of AGEs [8, 9]. For diabetic nephropathy patients, the increase in the concentration of AGEs in the body is not only due to the accelerated production of endogenous AGEs but also due to the decreased clearance of AGEs by the kidney [10, 11]. For diabetic nephropathy patients, the increase of AGEs in the body will further aggravate kidney damage, thus forming a vicious circle [12]. In addition, excessive oxidative stress, chronic inflammation, and uremia may all lead to an increase in the formation of AGEs, thus promoting the occurrence of AGEs and patient death [13–15]. Studies have shown that serum AGE concentration is negatively correlated with residual renal function and can be increased by 5 to 100 times in diabetic nephropathy patients [16, 17]. Reducing the level of chronic inflammatory response in diabetic patients is an efficient method to prevent and treat diabetic nephropathy [18].

Increasing the intake of dietary fiber can help maintain the stability of intestinal microecology [19]. Inulin is one of the most thoroughly studied prebiotics, which has remarkable effects in regulating intestinal flora and anti-inflammatory [20]. Studies have revealed that inulin can directly or indirectly regulate the composition and balance of intestinal microorganisms and promote the production of bioactive metabolites, such as short-chain fatty acids (SCFAs), amino acids, and peptides. Inulin could act on intestinal epithelial cells, participate in the regulation of immune cells and cytokines, defend against pathogen invasion, and enhance host immune response [21, 22]. Nevertheless, the regulatory effects of oral inulin on intestinal flora structure and microbial metabolic pathways in T2DM patients remain to be further studied.

T2DM is a chronic low-grade inflammatory disease, with the persistent low-grade inflammatory response in patients being an important feature [23]. Long-term low-grade inflammatory response and abnormal activation of the immune system are closely linked to the development of T2DM, and the elevated concentrations of systemic inflammatory markers (IL-1*β* and IL-6) are risk factors for complications of T2DM [24]. Inulin is a widely used prebiotic, which can selectively promote the growth of a variety of beneficial microorganisms in the host gut, regulate host immune function, and improve host health [25]. Human microecology is a complex and dynamically balanced ecosystem with more than 100 trillion symbiotic microorganisms, among which more than 1000 kinds of bacteria survive in the human gut, covering more than 50 bacterial categories, such as *Actinomyces*, *Proteobacteria*, *Bacteroidetes*, and lactobacilli. These complex microbial communities in the human gut are closely related to the mucosal immune system. The rich microbial species and a variety of immune cells on the surface of the human intestinal mucosa are the basis for regulating the systemic inflammatory response. Previous studies have shown that oral inulin can significantly change the abundance and quantity of host intestinal flora, as well as the type and quantity of metabolites of various intestinal microorganisms including SCFAs [26, 27]. SCFA is a class of intestinal microbiota metabolites with significant anti-inflammatory effects. It is a focus of current research to study the mechanism of this class of anti-inflammatory metabolites absorbed into the blood circulation through the intestinal mucosa and exerted through the “gut-kidney” axis in the treatment of diabetic kidney injury. However, at present, most of the studies on the health function of inulin are the analysis of intestinal flora, and there is still a lack of further studies on the regulatory substances and mechanisms of the “gut-kidney” axis.

For these reasons, based on a diabetic mouse model, this study investigated the effects of oral inulin on inflammation, AGEs, and intestinal SCFA production in mice and further investigated the mechanism of treatment of diabetic kidney injury by inulin through serum metabolomics. Our findings may provide a new view for clinical prevention and treatment of T2DM nephropathy.

## 2. Materials and Methods

### 2.1. High-Fat Diet and Streptozotocin-Induced Type 2 Diabetic Mice

Thirty-six 6-week-old healthy male Institute of Cancer Research (ICR) mice (weighing 18–22 g) were purchased from the Center of Comparative Medicine of Yangzhou University (Yangzhou, China). In order to avoid mice eating each other's feces during feeding, only one mouse was placed in each cage. The experiment was approved by the Ethics Committee of Jiangsu University (protocol code UJS-IACUC-AP-2021032009 and date of approval: January 2021). Inulin was purchased from Liaoning Zhongoccurrence Technology Co., LTD. (Dalian, China). The inulin polymerization degree is 2–60 (purity > 86.0%, water content ≤ 4.5%, ash ≤ 0.2%). To investigate the effect of oral inulin in the treatment of T2DM mice, we divided the mice into three groups, including the normal control (NC) group, T2DM (DM) group, and DM + inulin diet (INU) group, with 12 mice in each group. The T2DM diabetic mouse model was constructed using the method reported by Li et al. [28]. Experimental hyperglycemia was induced in mice by feeding them high-fat diet formula every day; all the mice in the DM and INU groups mice were given high-fat diets (product code: XTHF60-1, Nanjing Synergetic Biology, Nanjing, China) throughout the experiment, while NC group mice were given the ordinary diet (product code: 1010009, Nanjing Synergetic Biology, Nanjing, China) (Table 1). The energy supply ratios of protein, fat, and carbohydrate in a high-fat diet were 18.14%, 60.65%, and 21.22%, respectively. In comparison, the energy supply ratios of protein, fat, and carbohydrates in the general diet were 20.54%, 12.79%, and 66.67%, respectively. In the 5th week, mice in the DM and INU groups were injected intraperitoneally with streptozotocin (50 mg/kg) for 5 consecutive days to induce T2DM. Streptozotocin is dissolved with sodium citrate buffer (pH 4.2–45) in advance, streptozotocin solution is ready for use, and the use process is kept away from light. Each mouse was given an intraperitoneal injection of less than 1 mL [29]. The glucose concentration in the tail vein blood of the mice was measured by a blood glucose meter (Sanuo, Shanghai, China) every 5 days. A T2DM mouse model with fasting blood glucose (FBG) higher than 11.1 mmol/L was established successfully [29]. Inulin was given to mice by intragastric administration. The concentration of 50 g/L inulin solution was prepared with distilled water at 60°C and then cooled to room temperature for mouse gavage, ensuring that the amount of liquid for each mouse was less than 1 mL. At the beginning of the experiment, the INU group was given inulin at the dose of 500 mg/kg gavage daily until the end of the 12th week. All the mice in the 3 groups were fed until the end of the 12th week. After finishing the experimental periods, mice were euthanized by intraperitoneal injection of urethan (Sigma-Aldrich, St. Louis, MO, USA, 700 mg/kg), and blood, spleen, and kidney samples were collected [30].

### 2.2. Blood Lipids, SCFAs, and Oxidative Stress Levels Detection

The biochemical indices in mouse serum were determined by a semiautomatic biochemical analyzer (Shenzhen, China). The detection kits were purchased from Nanjing Jiancheng Bioengineering Institute (Nanjing, China), including FBG (A154-2-1), blood urea nitrogen (BUN, C013-2-1), triglycerides (TGs, A110-1-1), total cholesterol (TC, A111-1-1), low-density lipoprotein cholesterol (LDL-C, A113-1-1), malondialdehyde (MDA, A003-1-1), and superoxide dismutase (SOD, A001-3-2).

The three main SCFAs including acetic acid, propionic acid, and butyric acid detection in mouse serum were performed at Health Testing Center, Zhenjiang Center for Disease Control and Prevention (Zhenjiang, China) using liquid chromatography−mass spectrometry (LC-MS/MS). The sample derivatization process followed Ebert et al.'s reporting method [11]. The LC-MS/MS system consists of an UHPLC 1290 Infinity II System (Agilent Technologies, Santa Clara, CA, USA) coupled to a tandem mass spectrometer AB Sciex 4500 QTRAP (Sciex, Marsiling, Singapore) operated under positive ESI mode. The instrument parameters were CUR 30, GS1 50, GS2 60, capillary voltage 5.5 kV, and source temperature 550°C. Chromatographic separation was achieved on an ACQUITY UPLC BEH C18 1.7 *μ*m 2.1 × 100 mm (Waters Technologies, Wilmington, DE, USA) using as mobile phase (A) water +0.1% formic acid +5 mmol/L ammonium formate and (B) methanol. The elution program (%B) was 0–1.0 min 10%, 1.0–1.5 min 10–30%, 1.5–5.0 min 30–100%, 5.0–6.0 min 100%, 6.0–6.1 min 100–10%, and 6.1–8.0 min 10%. The flow rate was 0.3 mL/min, and the column temperature was 40°C.

### 2.3. Histological Analysis of Kidney Samples

Renal parenchymal tissue was fixed with 4% paraformaldehyde at room temperature for 24 h, then dehydrated by gradient concentration of ethanol, treated with xylene transparent, and embedded in paraffin. Samples were prepared by the Lecia slicing mechanism; the tissue section thickness was 4 *μ*m. The samples were stained by hematoxylin and eosin (H&E) and sealed with neutral gum. The morphological changes of renal parenchymal tissue were observed under an optical microscope (Lecia, Wetzlar, Germany). The kidney injury in mice was evaluated according to the methods reported by He et al. [31].

### 2.4. qRT-PCR Assay

The qPCR assay was based on the methods reported by Yan et al. [29]. Total RNA from the mouse spleen was extracted by RNA-easy Isolation Kit (R701, Vazyme, Nanjing, China), and cDNA was synthesized by reverse transcription kit (R312, Vazyme, Nanjing, China) for qPCR assay. AceQ Universal SYBR qPCR Master Mix Kit (Q511, Vazyme, Nanjing, China) was used in the qPCR assay. The total qPCR reaction system was 10 *μ*L, including 5 *μ*L of 2x SYBR Green Master premix (Vazyme, Nanjing, China), 0.2 *μ*L upstream and downstream primers (10 *μ*mol/L), and 1 *μ*L cDNA template. The qPCR reaction process consisted of 40 cycles, including initial denaturation at 95°C for 5 min, denaturation at 95°C for 3 s, annealing at 58°C for 20 s, and extension at 72°C for 30 s. Using GAPDH as an internal reference, the formula 2^−ΔΔCt^ was used to calculate the relative gene expression. The PCR primer sequence is shown in Table 2, which was synthesized by Suzhou GENEWIZ Company (Suzhou, China).

### 2.5. ELISA Assay

Serum levels of IL-1*β* and AGEs in mice were determined by ELISA. The IL-1*β* kit (catalog number: 3748) was purchased from Meimian Biotechnology Co., Ltd. (Yancheng, China), and the AGE kits (catalog number: XYEB353Ge) were purchased from Shanghai Enzyme-linked Biotechnology Co., Ltd. (Shanghai, China). The specific procedures were as follows: 50 *μ*L samples were incubated in the well at 37°C for 30 min and repeated washing three times to remove unbound samples; horseradish peroxidase-labeled goat anti-human IgG was added and incubated at 37°C for 30 min. The absorbance at 450 nm was measured by the enzyme-labeled instrument (Bio-Rad, Hercules, USA). The concentrations of IL-1*β* and AGEs were calculated using standard curves.

### 2.6. Plasma Sample Preparation and Untargeted Metabolomics Analysis

The pretreatment process and detection process of untargeted metabolomics samples were carried out according to Ebert et al.'s reported method [11]. The sample supernatant after treatment was transferred to a sample bottle with a liner tube for the UPLC-MS/MS detection and analysis. ACQUITY UPLC BEH C18 column was used in the UPLC-MS/MS system (Waters, Massachusetts, USA, 100 mm × 2.1 mm, 1.7 *μ*m). ESI electrospray ionization method and positive and negative ion modes were used for the collection. Peak identification, peak matching, peak alignment, and normalization were performed on the original UPLC-MS/MS data using Progenesis QI 2.3 software (Waters, Massachusetts, USA).

### 2.7. Statistical Analysis

The variable importance (VIP) score in the projection reflects the contribution of the analyzed variables to the OPLS-DA model. The potential differential metabolites were screened according to VIP > 1 and *p* < 0.05 rule. The obtained differential metabolites were retrieved and confirmed in the Human Metabolome Database (HMDB). Based on the Kyoto Encyclopedia of Genes and Genomes (KEGG) database, the related metabolic pathways of the potential differential metabolites were determined.

SSPS 22.0 statistical software was used for data analysis, and the data were expressed as mean ± standard deviation (SD). One-way analysis of variance was used for comparison among multiple groups. Statistical graphs were generated using GraphPad Prism 8 software, and *p* < 0.05 was considered statistically significant.

## 3. Results

### 3.1. Changes in Body Weight and Blood Biochemical Indexes of Mice

**Figure 1:**
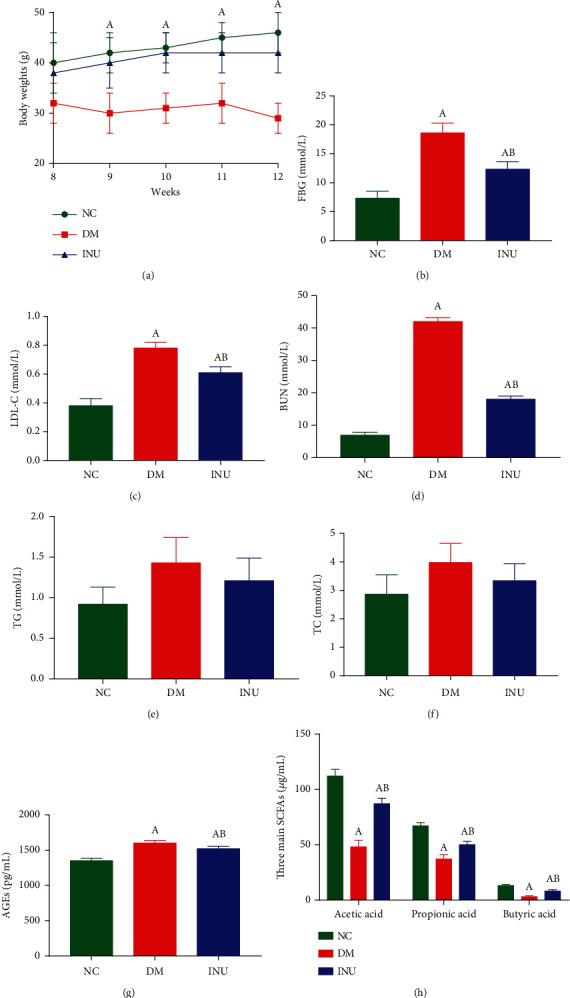
The change in body weight and blood biochemical indexes of mice. (a) Body weight. (b) Fasting blood glucose (FBG). (c) Low-density lipoprotein cholesterol (LDL-C). (d) Blood urea nitrogen (BUN). (e) Triglycerides (TGs). (f) Total cholesterol (TC). (g) Advanced glycation end products (AGEs). (h) Serum acetic acid, propionic acid, and butyric acid levels (the three main SCFAs). (A) Compared with the NC group, *p* < 0.05. (B) Compared with the DM group, *p* < 0.05.

From week 8 to week 12, the change in body weight and blood biochemical indexes of mice were shown in Figure 1(a). There was no significant difference in body weight between the NC and INU groups (*p* > 0.05), but the body weight of the DM group was significantly decreased compared with the NC and INU groups (*p* < 0.05). At the end of the experiment, compared with the NC group, the levels of FBG, LDL-C, and BUN in the DM and INU groups were significantly increased (*p* < 0.05); compared with the DM group, the blood biochemical indices in the INU group were significantly reduced (*p* < 0.05). There were no significant changes in TC and TG levels in the 3 groups (*p* > 0.05) (Figures 1(b)–1(f)). Compared with the NC group, the level of AGEs in the DM and INU groups was significantly increased (*p* < 0.05). Compared with the DM group, the level of AGEs in the INU group was significantly decreased (*p* < 0.05) (Figure 1(g)). Compared with the NC group, the concentrations of acetic acid, propionic acid, and butyric acid in the DM and INU groups were significantly decreased (*p* < 0.05). Compared with the DM group, the concentrations of acetic acid, propionic acid, and butyric acid in the INU group were significantly increased (*p* < 0.05) (Figure 1(h)).

### 3.2. Inulin Inhibits Inflammation and Oxidative Stress in Mice

**Figure 2:**
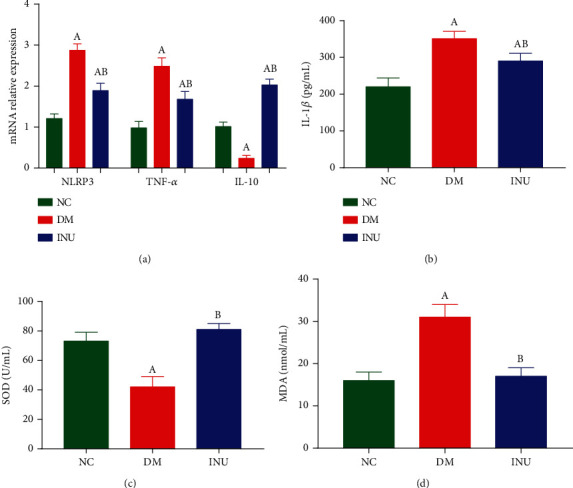
The expressions of inflammatory factors in the spleen and oxidative stress factors in serum. (a) The mRNA levels of inflammatory factors including NLR family pyrin domain-containing 3 (NLRP3), tumor necrosis factor (TNF)-*α,* and interleukin (IL)-10 in spleen tissues. (b) Interleukin (IL)-1*β*. (c) Superoxide dismutase (SOD). (d) Malondialdehyde (MDA). (A) Compared with the NC group, *p* < 0.05. (B) Compared with the DM group, *p* < 0.05.

Compared with the NC group, the mRNA expressions of the NLR family pyrin domain-containing protein 3 (NLRP3) and tumor necrosis factor-*α* (TNF-*α*) in spleen tissues of the DM and INU groups were significantly increased (*p* < 0.05). Compared with the DM group, the mRNA expressions of NLRP3 and TNF-*α* in the spleen tissue of the INU group were significantly decreased (*p* < 0.05). Compared with the NC group, the mRNA expression of interleukin-10 (IL-10) in the spleen tissue of the DM group was significantly decreased (*p* < 0.05), while the mRNA expression of the INU group was significantly increased (*p* < 0.05) (Figure 2(a)). Compared with the NC group, serum interleukin-1*β* (IL-1*β*) level in the DM and INU groups was significantly increased (*p* < 0.05); compared with the DM group, serum IL-1*β* level in the INU group was significantly decreased (*p* < 0.05). Compared with the NC group, the serum SOD level in the DM group was significantly decreased (*p* < 0.05). Compared with the DM group, the SOD level of the INU group was significantly increased (*p* < 0.05). Compared with the NC group, the serum MDA level in the DM group was significantly increased (*p* < 0.05). Compared with the DM group, the serum MDA level in the INU group was significantly decreased (*p* < 0.05) (Figures 2(b)–2(d)).

### 3.3. Inulin Reduces Kidney Injury in T2DM Mice

**Figure 3:**
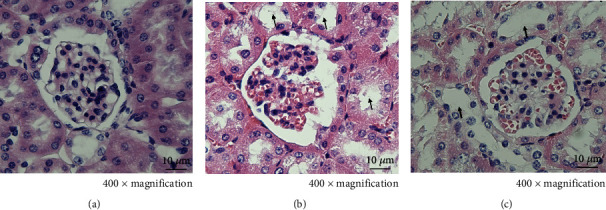
Histopathological analysis of renal parenchymal tissue in different groups with H&E staining at 400x magnification. (a) NC group. The structure of kidney bodies was complete, and the renal follicular cavity was visible, without hyperplasia and other lesions. (b) DM group. The glomerular volume and mesangial matrix increased, and renal tubular epithelial cells vacuolated (↑). (c) DM + inulin diet (INU group). The epithelial cells of renal tubules shed less, and the types of renal tubules decreased (↑).

After H&E staining, it was found that the structure of kidney bodies in the NC group was complete, and the renal follicular cavity was visible, without hyperplasia and other lesions. In the DM group, glomerular volume and mesangial matrix increased, renal tubular epithelial cells vacuolated, and part of renal tubular epithelial cells shed to form renal tubular type. In the INU group, the epithelial cells of renal tubules shed less, and the types of renal tubules decreased (Figure 3).

### 3.4. Inulin Affects the Constitution of Plasma Metabolites

**Figure 4:**
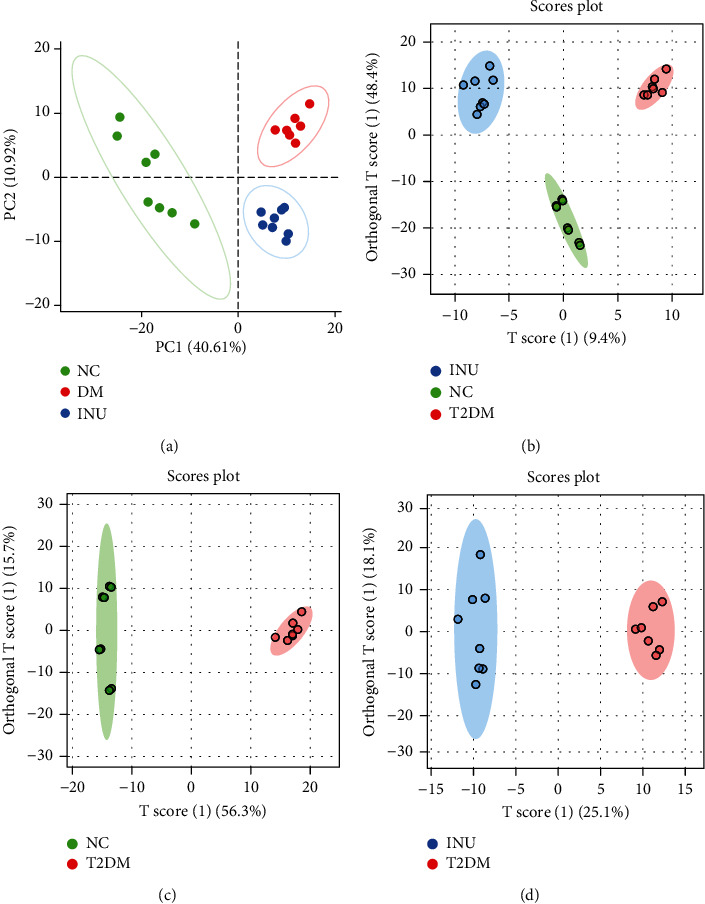
The (a) PCA and (b–d) OPLS-DA score plot. The NC group (green), the DM group (red), and the INU group (blue) (*n* = 8/group). “(1)” represents that this is the first set of analysed data.

The metabolomics of the NC, DM, and INU groups was analyzed by the UPLC-MS/MS technique. Nine hundred twenty-six metabolites were detected in the ESI− mode. The principal component analysis (PCA) showed that the plasma samples of the three groups were well separated in ESI− mode. The samples in the DM group were separated from those in the NC group, indicating that the endogenous metabolites in mice induced by T2DM had significant changes. Samples in the INU group were also significantly separated from those in the DM group and were closer to those in the NC group than those in the DM group, indicating that inulin intervention had a significant reversal effect on metabolic disorders induced by T2DM (Figure 4(a)). Then, the OPLS-DA model was used to analyze the differences among the three groups. It could be seen that in ESI+ mode and ESI− mode, the samples of the NC, DM, and INU groups were well clustered, and the three groups of samples were obviously separated. In ESI+ mode, the parameters of the OPLS-DA model were *R*^2^*X* = 0.59, *R*^2^*Y* = 0.989, and *Q*^2^ = 0.968. In ESI− mode, the parameters of the OPLS-DA model were *R*^2^*X* = 0.827, *R*^2^*Y* = 0.988, and *Q*^2^ = 0.959. The closer *R*^2^ is to 1, the more stable the model is. *Q*^2^ > 0.5 indicates that the model has good predictive ability (Figure 4(b)). Next, the differential metabolites among each group were evaluated using variable influence on the project score (VIP > 1) of OPLS-DA and Student's *t*-test analysis (with *p* < 0.05) (Figures 4(c) and 4(d)). The variation trends of the above differential metabolites are shown in Tables 3 and 4. Compared with the NC group, the upregulated metabolites in the DM group were L-acetylcarnitine, prostaglandin F1a, L-palmitoylcarnitine, prostaglandin H2, leukotriene A4, taurocholic acid, pyridoxine 5′-phosphate, L-tryptophan, L-carnitine, and allodeoxycholic acid; the downregulated metabolites were dUMP, gamma-tocotrienol, phosphatidylcholine (20:4(5Z,8Z,11Z,14Z)/18:0), GlcCer (d18:1/16:0), phosphatidyl ethanolamine (20:2(11Z,14Z)/16:1(9Z)), phosphatidylcholine (16:0/0:0), and L-lactic acid. Compared with the DM group, the upregulated metabolites in the INU group were glycocholic acid, mevalonic acid, tryptophanol, isoleucine, phosphatidylcholine (16:0/0:0), phosphatidyl ethanolamine (20:2(11Z,14Z)/16:1(9Z)), 2-phenylethanol glucuronide, and pregnanetriolone; and the downregulated metabolites were glucosylsphingosine, prostaglandin H2, leukotriene A4, and pyridoxine 5′-phosphate.

### 3.5. Inulin Affects Plasma Metabolic Pathway

**Figure 5:**
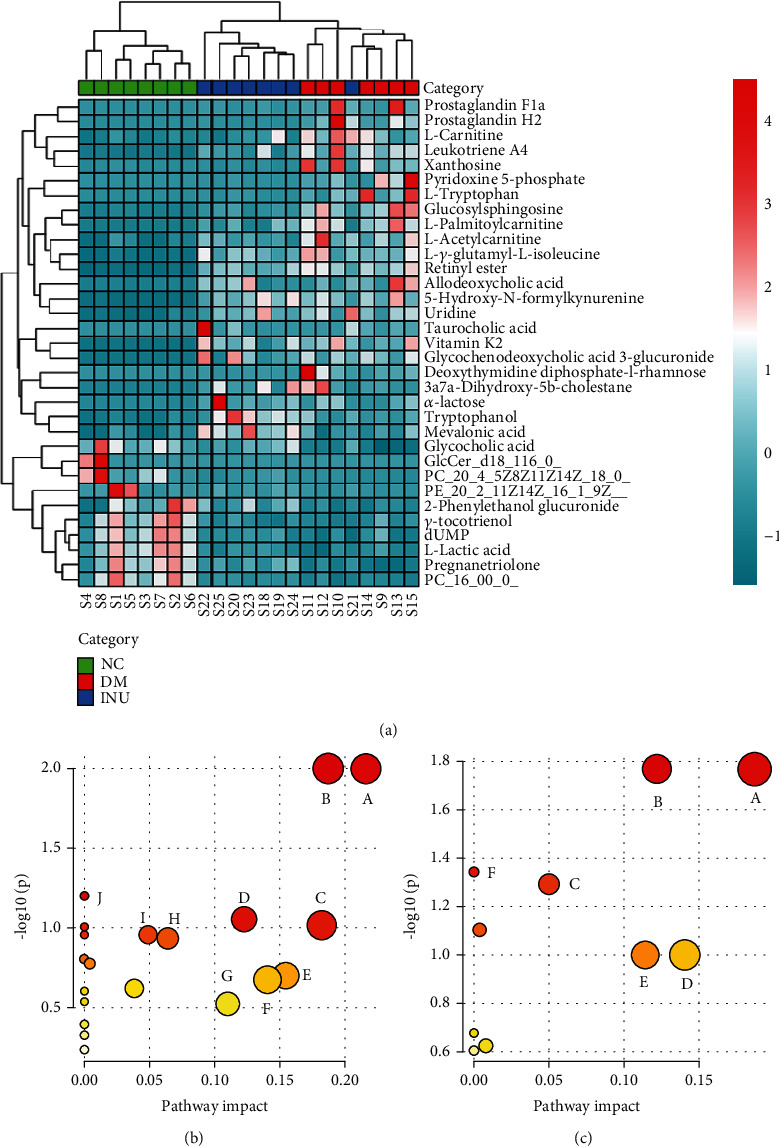
The cluster heat map and metabolic pathway analysis (*n* = 8/group). (a) Cluster heat map for the NC group (green), the DM group (red), and the INU group (blue). (b) The metabolic pathway analysis of plasma metabolites in the NC and DM groups. (A) Glycerophospholipid metabolism, (B) arachidonic acid metabolism, (C) tryptophan metabolism, (D) pyrimidine metabolism, (E) retinol metabolism, (F) pentose and glucuronate interconversions, (G) galactose metabolism, (H) primary bile acid biosynthesis, (I) vitamin B6 metabolism, and (J) linoleic acid metabolism. (c) The metabolic pathway analysis of plasma metabolites in the DM and INU groups. (A) Arachidonic acid metabolism, (B) glycerophospholipid metabolism, (C) vitamin B6 metabolism, (D) pentose and glucuronate interconversions, (E) terpenoid backbone biosynthesis, and (F) valine, leucine, and isoleucine biosynthesis.

The identified metabolites were input into the MetaboAnalyst database to construct a KEGG analysis of the metabolic pathway and set the critical value of the influence value of the metabolic pathway as 0.1. If the value is higher than this, it will be regarded as a potential target pathway (Figure 5(a)). Compared to the NC group, multiple plasma metabolic pathways were significantly altered in the DM group mice, including glycerophospholipid metabolism, arachidonic acid metabolism, tryptophan metabolism, pyrimidine metabolism, retinol metabolism, pentose and glucuronate interconversions, galactose metabolism, primary bile acid biosynthesis, vitamin B6 metabolism, and linoleic acid metabolism (Figure 5(b)). Compared to the DM group, multiple plasma metabolic pathways were significantly altered in the INU group mice, including arachidonic acid metabolism, glycerophospholipid metabolism, vitamin B6 metabolism, pentose and glucuronate interconversions, terpenoid backbone biosynthesis, and valine, leucine, and isoleucine biosynthesis (Figure 5(c)).

## 4. Discussion

T2DM is a metabolic disease mainly characterized by elevated blood glucose concentration, and its onset is mainly related to genetic and environmental factors [32, 33]. It is a chronic low-grade inflammatory disease, with a persistent low-grade inflammatory response in patients being an important feature [23, 34]. People susceptible to T2DM all have adverse factors such as aging and overnutrition, which can activate the innate immune system, induce macrophages and adipocytes in the body to secrete a variety of inflammatory factors, and further cause insulin resistance and insulin secretion dysfunction [35–37]. Long-term low-grade inflammatory response and abnormal activation of the immune system are closely related to the development of T2DM, and elevated concentrations of systemic inflammatory markers (IL-1*β* and IL-6) are risk factors for complications of T2DM [38]. Studies have shown that oral high-dose anti-inflammatory drug aspirin can significantly reverse insulin resistance, hyperglycemia, and other symptoms in patients with T2DM [39]. These findings make people realize that inflammatory response is related to the development of diabetes and insulin resistance, and anti-inflammatory therapy is an important means to alleviate diabetes and its complications.

In this study, it was found that the inflammatory response and oxidative stress in T2DM mice were significantly increased, and the expressions of inflammatory factors NLRP3 and TNF-*α* were significantly increased. Nucleotide-binding oligomerization domain-like receptor protein (NLRP) has many subtypes, such as NLRP1, NLRP3, NLRP6, and NLRC4 [40]. Our findings and those of other researchers show that NLRP3 inflammasome is one of the most studied cytoplasmic inflammasomes, and it plays a key role in the innate immune defense after infection by bacteria, fungi, and viruses, and its overactivation can participate in the occurrence and development of a variety of chronic diseases [41, 42]. Studies have shown that the activation of NLRP3 inflammasome in the liver, skeletal muscle, and other tissues can significantly increase the risk of T2DM [43, 44]. It is thought that activation of the NLRP3 inflammasome is central to the pathophysiology of obesity and insulin resistance [45]. The activation of NLRP3 inflammasome in the liver, skeletal muscle, and adipose tissue secretes a large amount of mature IL-1*β* [46]. High levels of IL-1*β* will lead to insensitivity to insulin in obese individuals [47]. Activated IL-1*β* can directly damage pancreatic *β* cells on the one hand; and on the other hand, continuous cascade inflammation will aggravate the damage and death of *β* cells [48]. However, after weight loss in obese T2DM patients, the expressions of IL-1*β* and NLRP3 inflammasome are decreased, and the FBG and insulin resistance in T2DM patients are also decreased [49]. Diabetic nephropathy has become one of the public problems seriously endangering human health in the world, accounting for 30%–50% of end-stage nephropathy [50]. Previous studies have shown that long-term hyperglycemic metabolic disorders and hemodynamic disorders in vivo are the main pathogenesis of diabetic nephropathy, but now more and more researchers have proposed that the inflammatory mechanism is the key factor in the progression of the disease [51]. When AGEs produced in the body enter the blood circulation, they cause changes in renal hemodynamics and neurohumoral regulation of the body and then lead to inflammation [52]. In hyperglycemia, the body can produce a variety of damage-associated molecular patterns, such as fatty acids and reactive oxygen species, which can be recognized by relevant pattern recognition receptors and cause pyrosis [53]. In addition, high levels of IL-1*β* in diabetic patients can also induce pyrodeath of renal foot process cells, and the level of IL-1*β* in diabetic nephropathy patients is closely related to the level of urinary protein and the time of progression to end-stage renal disease [54].

Inulin is a widely used prebiotic, which can selectively promote the growth of a variety of beneficial microorganisms in the host gut, regulate host immune function, and improve host health [55]. Human microecology is a complex and dynamically balanced ecosystem, with more than 100 trillion symbiotic microorganisms, among which more than 1000 kinds of bacteria survive in the human gut, covering more than 50 bacterial categories, including *Actinomyces*, *Proteobacteria*, *Bacteroidetes*, and lactobacilli [56]. These complex microbial communities in the human gut are closely related to the mucosal immune system [57]. The surface area of human intestinal mucosa far exceeds the surface area of the human body. The rich microbial species and a variety of immune cells on the surface of the human intestinal mucosa are the basis for regulating systemic inflammatory response. Previous studies have shown that oral inulin can significantly change the abundance and quantity of host intestinal flora, as well as the type and quantity of metabolites of various intestinal microorganisms [26]. These metabolites enter the body through blood circulation and play various physiological functions through the “intestine-organ” axis [58]. Our study shows that inulin can significantly increase the production of SCFAs, an important metabolite of intestinal flora. SCFAs are a series of organic acids with less than 6 carbon atoms, mainly acetic acid, propionic acid, and butyric acid, which are obtained by anaerobic bacteria in the colon by fermenting carbohydrates that cannot be digested by humans, accounting for more than 90% of the total SCFAs. SCFAs in the colon can not only provide energy for intestinal mucosal cells and promote cell metabolism and growth but also reduce the environmental pH in the colon, reduce the growth of harmful bacteria, and prevent intestinal dysfunction [59]. More importantly, recent studies have confirmed that SCFAs can inhibit the production of anti-inflammatory factors and play an adjuvant role in the treatment of a variety of chronic inflammatory diseases [60]. However, there is still a lack of studies on reducing the chronic inflammatory response in diabetic patients and treating complications such as kidney injury through oral inulin.

In this study, it was found that the inflammatory response of T2DM mice was significantly higher than that of normal mice, the chronic inflammatory response was significantly inhibited, and the degree of kidney injury was significantly improved after oral inulin administration. Metabolomics analysis showed that the contents of various metabolites related to fatty acid metabolism and inflammatory response were abnormal in the plasma of T2DM mice. Studies have shown that the level of L-acetylcarnitine in diabetic patients was a specific diagnostic indicator of diabetic complications, and the large accumulation of L-acetylcarnitine in plasma may imply impaired mitochondrial function and fatty acid oxidation in diabetic patients [61]. L-Palmitoylcarnitine is a long-chain acylcarnitine, which is the product of fatty acid metabolism. The increased concentration of L-palmitoylcarnitine is positively correlated with chronic inflammation in patients with T2DM and is associated with abnormal fatty acid metabolism and fatty acid biosynthesis in mitochondria [62]. A metabolic marker of lactoylation associated with chronic inflammatory responses in the body. In diabetes, lactyl-CoA is a marker of lacacylation metabolism and is associated with chronic inflammation in vivo due to the obstruction of the glycolysis pathway. In T2DM patients, the glycolytic pathway is blocked, resulting in the inhibition of lacacylation metabolism, namely, the decrease of lactyl-CoA concentration [63]. Prostaglandin H2 is a prostaglandin extracted from arachidonic acid, which can be further derived into other prostaglandins and thrombinins in vivo. Elevated prostaglandin H2 levels in T2DM patients are associated with a chronic inflammatory response in vivo [64]. In addition, high plasma concentration of leukotriene A4 has been associated with chronic low-grade inflammation in patients with T2DM and with the development of diabetic neuropathy [65, 66]. L-Carnitine is a highly effective antioxidant. Patients with T2DM can resist oxidative stress and apoptosis caused by a large number of free radicals by enhancing L-carnitine in vivo. Moreover, it can effectively inhibit the depolarization and permeability enhancement of mitochondrial membrane and reduce cell apoptosis by transferring long-chain fatty acids accumulated around mitochondria [67]. We also found significant changes in the concentrations of many lipid metabolites in T2DM mice, including phosphatidylcholine (20:4(5Z,8Z,11Z,14Z)/18:0), GlcCer (d18:1/16:0), phosphatidyl ethanolamine (20:2(11Z,14Z)/16:1(9Z)), and phosphatidylcholine (22:2(13Z,16Z)/14:0). As T2DM patients are more likely to convert lipids into smaller molecules, the levels of phosphatidylcholine and phosphatidyl ethanolamine are reduced, which also leads to the higher risk of cardiovascular diseases in T2DM patients [68].

After inulin treatment in T2DM mice, we found significant changes in plasma concentrations of multiple metabolites. Glucosylsphingosine is the metabolite of amino acid and sphingolipid, and the potential disturbance of amino acid and sphingolipid metabolism is associated with the occurrence and severity of T2DM [69]. In this study, it was found that the concentration of glucosylsphingosine in the plasma of mice was decreased after oral administration of inulin, suggesting that inulin had a therapeutic effect on T2DM. We also found that the concentration of several inflammation-related metabolites decreased, including prostaglandin H2 and leukotriene A4, and that proinulin could significantly reduce the inflammatory response in T2DM mice. Phytosphingosine is also an inflammatory response-related metabolite, and its concentration in patients decreased with the improvement of T2DM and the reduction of chronic inflammation [70]. In this study, we found that inulin can also downregulate the level of phytosphingosine in T2DM mice, suggesting the anti-inflammatory effect of inulin. Increased concentration of pyridoxine 5′-phosphate in plasma, a marker of renal insufficiency in patients with diabetes, is associated with renal insufficiency in patients with T2DM [71]. Our results suggest that oral inulin can significantly produce pyridoxine 5′-phosphate in T2DM mice, which is consistent with the effect of inulin in improving kidney injury in T2DM mice.

## 5. Conclusion

Oral inulin emerges as a promising treatment option for diabetes and kidney injury. Inulin treatment can significantly increase the production of the three main SCFAs in the intestine and reduce the concentration of proinflammatory factor AGEs and inflammation levels in the body. Further metabolomics studies showed that inulin may be induced by glycerophospholipid metabolism, taurine and hypotaurine metabolism, arginine biosynthesis, and tryptophan metabolism affected the inflammatory response in T2DM mice.

## Figures and Tables

**Table 1:** The ingredients of general diet and high-fat diet.

**Ingredients**	**Ordinary diet**	**High-fat diet**
Crude protein	18. 8%	22.9%
Crude fat	5.2%	34.1%
Coarse fiber	3%	3%
Coarse ash	5.4%	4.4%
Calcium	1.1%	1.1%
Total phosphorus	0.7%	0.7%

**Nutrient composition**	**Energy supply ratio**	
Protein	20%	20%
Carbohydrate	70%	20%
Fat	10%	60%
Total kcal/g	4056.8 kcal/1055 gm = 3.84	4057 kcal/773.8 gm = 5.24

**Table 2:** The primers sequence of qRT-PCR assay.

**Genes**	**Primer sequences (**5′**⟶**3′**)**
*Mum_GAPDH*	F: AATGGATTTGGACGCATTGGT R: TTTGCACTGGTACGTGTTGAT

*Mum_TNF-α*	F: GAACTGGCAAAAGGATGGTGA R: TGTGGGTTGTTGACCTCAAAC

*Mum_NLRP3*	F: ATTACCCGCCCGAGAAAGG R: CATGAGTGTGGCTAGATCCAAG

*Mum_IL-10*	F: GAAGCTCCCTCAGCGAGGACA
	R: TTGGGCCAGTGAGTGAAAGGG

**Table 3:** Potential differential metabolites between the NC group and the DM group.

**Metabolites**	**Formula**	**HMDB**	**m/z**	**Retention time (min)**	**Change**
Uridine	C_9_H_12_N_2_O_6_	HMDB00296	243.06	1.75	↑
Vitamin K2	C_41_H_56_O_2_	HMDB30017	625.42	9.85	↑
L-Acetylcarnitine	C_9_H_17_NO_4_	HMDB00201	202.11	4.33	↑
Prostaglandin F1a	C_20_H_36_O_5_	HMDB02685	377.23	4.94	↑
L-Palmitoylcarnitine	C_23_H_45_NO_4_	HMDB00222	436.28	6.25	↑
5-Hydroxy-N-formylkynurenine	C_11_H_12_N_2_O_5_	HMDB04086	287.05	1.93	↑
Prostaglandin H2	C_20_H_32_O_5_	HMDB01381	351.22	4.94	↑
dUMP	C_9_H_13_N_2_O_8_P	HMDB01409	307.03	1.3	↓
Leukotriene A4	C_20_H_30_O_3_	HMDB01337	317.21	6.05	↑
Gamma-tocotrienol	C_28_H_42_O_2_	HMDB12958	409.31	6.57	↓
Phosphatidylcholine (20:4(5Z,8Z,11Z,14Z)/18:0)	C_46_H_84_NO_8_P	HMDB08431	854.59	23.43	↓
Taurocholic acid	C_26_H_45_NO_7_S	HMDB00036	514.28	4.7	↑
GlcCer (d18:1/16:0)	C_40_H_77_NO_8_	HMDB04971	736.52	21.72	↓
Phosphatidyl ethanolamine (20:2(11Z,14Z)/16:1(9Z))	C_41_H_76_NO_8_P	HMDB09287	786.53	6.18	↓
Deoxythymidine diphosphate-l-rhamnose	C_16_H_26_N_2_O_15_P_2_	HMDB06354	529.06	4.49	↑
Pyridoxine 5′-phosphate	C_8_H_12_NO_6_P	HMDB01319	497.07	4.27	↑
L-Tryptophan	C_11_H_12_N_2_O_2_	HMDB00929	453.18	5.06	↑
L-Carnitine	C_7_H_15_NO_3_	HMDB00062	381.23	5.39	↑
Phosphatidylcholine (16:0/0:0)	C_24_H_50_NO_7_P	HMDB10382	530.3	6.62	↓
Allodeoxycholic acid	C_24_H_40_O_4_	HMDB00478	391.28	5.69	↑
Xanthosine	C_10_H_12_N_4_O_6_	HMDB00299	283.07	2.16	↑
Alpha-lactose	C_12_H_22_O_11_	HMDB00186	377.09	0.92	↑
3a,7a-Dihydroxy-5b-cholestane	C_27_H_48_O_2_	HMDB06893	449.36	22	↑
Glycochenodeoxycholic acid 3-glucuronide	C_32_H_51_NO_11_	HMDB02579	660.31	5.29	↑
L-Lactic acid	C_3_H_6_O_3_	HMDB00190	89.02	1.28	↓
Retinyl ester	C_20_H_30_O_2_	HMDB03598	301.22	5.46	↑

**Table 4:** Potential differential metabolites between the DM group and the INU group.

**Metabolites**	**Formula**	**HMDB**	**m/z**	**Retention time (min)**	**Change**
Glucosylsphingosine	C_24_H_47_NO_7_	HMDB00596	496.3	5.36	↓
Glycocholic acid	C_26_H_43_NO_6_	HMDB00138	500.28	5.75	↑
Mevalonic acid	C_6_H_12_O_4_	HMDB00227	129.06	3.89	↑
Tryptophanol	C_10_H_11_NO	HMDB03447	206.08	4.4	↑
Isoleucine	C_12_H_25_N_3_O_3_	HMDB00172	776.56	23.31	↑
Phosphatidylcholine (16:0/0:0)	C_24_H_50_NO_7_P	HMDB10382	530.3	6.62	↑
Prostaglandin H2	C_20_H_32_O_5_	HMDB01381	351.22	4.94	↓
Leukotriene A4	C_20_H_30_O_3_	HMDB01337	317.21	6.05	↓
Phosphatidylcholine (20:2(11Z,14Z)/16:1(9Z))	C_41_H_76_NO_8_P	HMDB09287	786.53	6.18	↑
Pyridoxine 5′-phosphate	C_8_H_12_NO_6_P	HMDB01319	497.07	4.27	↓
2-Phenylethanol glucuronide	C_14_H_18_O_7_	HMDB10350	655.23	3.16	↑
Pregnanetriolone	C_21_H_34_O_4_	HMDB41997	371.22	12.76	↑

## Data Availability

The paper data used to support the findings of this study are available from the corresponding authors upon request. Samples of the inulin are available from the authors.
